# The influence of amniotic membrane proteins on corneal regeneration when delivered directly or using hydrogel platforms

**DOI:** 10.3389/fmed.2025.1498319

**Published:** 2025-02-18

**Authors:** Ayla Basasoro, Javier Mendicute, Marta Rezola, Jorge Burgos, Mercedes Fernández, David Esporrín-Ubieto, Ana Sonzogni, Marcelo Calderón, Itxaso Calafel, Aitor Díaz, Juliana De Souza, Ana Aiastui, Mikel Azkargorta, Félix Elortza, Elena Vecino, Arantxa Acera

**Affiliations:** ^1^Hospital Universitario Donostia, Donostia-San Sebastián, Spain; ^2^Biogipuzkoa Health Research Institute, Donostia-San Sebastián, Spain; ^3^Hospital Universitario Cruces, Barakaldo, Spain; ^4^POLYMAT, Department of Polymers and Advanced Materials: Physics, Chemistry and Technology, Faculty of Chemistry, University of the Basque Country UPV/EHU, Donostia-San Sebastián, Spain; ^5^POLYMAT, Applied Chemistry Department, Faculty of Chemistry, University of the Basque Country UPV/EHU, Donostia-San Sebastián, Spain; ^6^NTEC (UNL-CONICET), Güemes 3450, Santa Fe, Argentina; ^7^IKERBASQUE, Basque Foundation for Science, Bilbao, Spain; ^8^CIDETEC, Basque Research and Technology Alliance (BRTA), Donostia-San Sebastián, Spain; ^9^Proteomics Platform, CIC bioGUNE, Basque Research and Technology Alliance (BRTA), CIBERehd, Derio, Spain; ^10^Department of Cell Biology and Histology, Experimental Ophthalmo-Biology Group (GOBE), University of the Basque Country UPV/EHU, Leioa, Spain

**Keywords:** hydrogel inserts, cornea, wound healing, rabbit, amniotic membrane

## Abstract

**Background/aims:**

Chemical burns to the eye cause extensive ocular surface damage, often resulting in permanent visual impairment. The amniotic membrane (AM), with its regenerative properties, has shown great potential in treating such injuries. Recent innovations, including hydrogel-based delivery systems, have been developed to improve drug retention and promote corneal healing. This study aimed to evaluate whether hydrogels loaded with proteins from human AM could enhance corneal wound healing effectively.

**Methods:**

Alkaline burns (8 mm diameter) were induced in the cornea of anesthetized male New Zealand White rabbits (*n* = 44) by placing filter paper soaked in 1M NaOH on them for 60 s. After rinsed the corneas immediately with a balanced salt solution, the wounds then received: (1) no treatment; (2) AM transplantation; or (3) a dynamic hyaluronic acid hydrogel based on gold thiolate loaded with an AM protein extract (AME); or (4) a physically cross-linked ocular hydrogel insert loaded with the same AME. The contralateral uninjured eye served as a control. The wound area and proportion of healed corneas was assessed in microphotographs. In addition, corneal histology was evaluated by hematoxylin–eosin and Masson’s trichrome staining, examining epithelial and stromal thickness, the endothelial layer, and inflammatory infiltration in the early (day 2) and late (day 28) phases of healing.

**Results:**

There was more frequent corneal wound closure in animals treated with the hydrogels (treatments 3 and 4) on day 14 (44.4 and 55.5%, respectively) than in the untreated controls (33.3%). Histologically, abnormal re-epithelialization and altered epithelial junctions were observed, with no significant differences in epithelial thickness. Endothelial damage correlated with significant thinning (*p* = 0.001), with treatments 2 and 3 producing significant differences in the inflammatory infiltrate (*p* = 0.01).

**Conclusion:**

Applying new biocompatible hydrogels to the ocular surface that release AM proteins may help close corneal wounds caused by caustic burns. The aggressive nature of burns hinders the detection of differences in the wound area between treatments 28 days after injury. However, improving the adhesion of a solid hydrogel to the mucosa of the ocular surface could enhance these outcomes by keeping the implant in contact with the wound for a longer period.

## 1 Introduction

Chemical eye burns produce considerable damage to the ocular surface epithelium, cornea and anterior segment, provoking permanent unilateral or bilateral visual impairment (1). These injuries may be caused by direct exposure of the eye to corrosive chemicals, and they may have severe consequences on the structure and function of the eyeball, and/or its surrounding tissues. The severity of an ocular chemical burn depends on various factors, including the nature of the chemical agent, the duration of exposure, the area exposed, the concentration of the agent and the promptness of treatment (2). Proper management of chemical eye burns requires a rapid and accurate initial assessment, followed by therapeutic interventions that minimize tissue damage, preventing the progression of the injury and promoting healing of the ocular surface (2). In this sense, the amniotic membrane (AM) has been proposed as a promising tool due to its unique composition and regenerative properties (3). This tissue is derived from the inner layer of the amniotic sac and its unique characteristics make it ideal for various therapeutic applications related to ocular conditions (4). There is clinical evidence that AM transplantation promotes corneal epithelial healing (5), reduces scar formation (6), suppresses inflammation (7) and inhibits angiogenesis (8, 9). Many of these effects have been attributed to the protein content of the AM, which includes growth factors (10), cytokines and structural proteins (11) that can modulate immune responses and promote tissue regeneration. The proteins obtained from AM preparations are influenced by several factors, including gestational and donor age (12), the method of preservation employed (13), as well as the filtration and centrifugation protocols used to prepare eye drops. Previously, our group characterized some growth factors in the AM known to be involved in re-epithelialization, such as beta-Fibroblast Growth Factor (bFGF), Endothelial Growth Factor (EGF), Hepatocyte Growth Factor (HGF), Nerve Growth Factor (NGF) and the endogenous anti-angiogenic factor endostatin (14). However, considering the potential of the AM in this process, it would be of interest to obtain a more comprehensive understanding of its protein profile.

Corneal ulcers are generally treated using eye drops, which requires frequent administration as the active ingredient only remains in contact with the ocular surface briefly due to the constant flushing by tears through blinking. Over the past decade, novel approaches to overcome this limitation have been developed, improving drug residence times on the cornea. These include the use of nanoparticles (15), liposomes (16), micro emulsions (17), Nano suspensions (18), polymeric micelles (19, 20) and hydrogel-based delivery systems (21). Hydrogels are three-dimensional polymeric structures made up of water embedded in polymeric chains that are designed to retain the cohesive properties of solids and the transport properties of liquids (22). Hydrogels can be broadly categorized into physical and chemical hydrogels based on the interactions establishing their network structure. Physical Hydrogels are mainly formed through physical interactions like hydrogen bonding, hydrophobic interactions or physical entanglement of polymer chains. These interactions are reversible and can be disrupted by altering environmental conditions, such as temperature, pH or ionic strength (23). Of these, hydrogels based on Eudragits® are of particular interest as combining this polymer with other biocompatible polymers (e.g., polyvinylpyrrolidone (PVP), polyvinyl alcohol (PVA), or polyethylene glycol (PEG)) enables some properties of the hydrogels to be modulated, such as their porosity, stability and cargo release capacity (24, 25). Significantly, the use of Eudragits has been explored in physically cross-linked hydrogels designed for ophthalmic applications (26–29).

Covalently crosslinking polymer chains, stabilizing the bonds in the hydrogel network, form chemical hydrogels. Such crosslinking typically involves chemical reactions like polymerization, crosslinking agents or enzymatic reactions (23). As a result, chemical hydrogels have stable lattice structures that are irreversible under normal conditions. However, modulating the degree of entanglement/cross-linking in these hydrogels can yield soft, degradable and permeable structures, with mechanical properties similar to those of tissues, thereby offering a promising solution for prolonged drug delivery and tissue engineering applications (25). Another key factor that enhances the potential for hydrogels to be used in clinical applications is their mucoadhesivity. The water content and the soft, flexible nature of hydrogels make them suitable to associate with mucin, through either electrostatic interaction, hydrogen bonding, hydrophobic interactions or Van der Waals forces. As a result, there have been efforts to develop hydrogels that adhere to mucous membranes with the potential to extend drug residence times (30). Recent years have seen the development of the next generation of hydrogels based on reversible bonds, namely dynamic hydrogels, offering the promise of being more adaptable and of inducing self-healing in natural tissues. These hydrogels possess a network of polymer chains that can form dynamic bonds that enable the material to reorganize its structure in response to external stimuli (31). Of particular interest are those gels based on interactions established between Au (I) or Ag (I) ions and thiolate polymers. These reversible interactions, namely, metal-thiolate (M-S) and disulphide (S-S) bonds provide gels that adopt features of an injectable material with permanent exchange ratios and self-healing capacities. In addition, if thiolate hyaluronic acid (HA-SH) is used, better mucoadhesivity to the cornea is expected thanks to the ability of thiol groups to react with the thiol groups of cysteine residues in the tear film’s mucin (30, 32).

This study set out to evaluate the efficacy of two hydrogel formulations for the treatment of corneal ulcers: one dynamic and injectable; and the other based on Eudragit combined with biocompatible polymers. This is a preliminary study in which, after optimizing the corresponding formulations, biocompatible hydrogels were obtained that could establish a baseline on which further improvements can be made. AM proteins were incorporated into the hydrogels to compare the rate of corneal ulcer closure between them, comparing their effects with the more traditional transplantation of AM fragments using sutures (AMT). Indeed, the AM protein extracts (AMEs) were analyzed out with a view to obtain new insights into the key molecules responsible for the therapeutic effects of the AM. This information may well aid the selection of specific proteins to be included in hydrogels, enhance current formulations and develop new, more effective treatment strategies.

## 2 Materials and methods

### 2.1 Amniotic membrane protein extraction

AMs were obtained from the Gynaecology and Obstetrics Department at Cruces University Hospital (Barakaldo, Spain), their use in this study was approved by the Cruces Ethics Committee (CEIC code E20/05). All human tissues were handled in accordance with the tenets of the Declaration of Helsinki regarding medical research involving human subjects. The patient selection process followed the same criteria established for AM donation intended for ocular surface surgery. The inclusion criteria required participants to be between 18 and 42 years of age, seronegative for human immunodeficiency virus (HIV), hepatitis B and C, and syphilis, and to have experienced full-term pregnancies (38–42 weeks). Exclusion criteria ruled out individuals with gestational or fetal diseases.

Human placentas were managed in alignment with the principles outlined in the Declaration of Helsinki. Following the acquisition of informed consent, four human placentas from Caucasian women were collected following elective caesarean deliveries and promptly processed under sterile conditions. First, the placentas were rinsed with sterile saline solution to eliminate blood clots. The AM was then meticulously detached from the chorion using blunt dissection and thoroughly rinsed several times in a saline solution enriched with antibiotics and antifungal agents (penicillin 10,000 U/mL, streptomycin 50 mg/mL, and amphotericin B 2.5 mg/mL). Subsequently, each AM was transferred to a sterile container containing a 1:1 mixture of Dulbecco’s Modified Eagle’s Medium (DMEM) and glycerol (Gibco®, USA) and preserved at −80°C until required for use.

The protocol for extracting proteins from the AM was carried out as established previously (14).

### 2.2 Proteomic analysis of the AM

Label-free protein identification based on liquid chromatography-mass spectrometry (LC–MS) was performed to assess the differences in the proteome of the AM tissue used for transplantation (directly in contact with the corneal epithelium) and the AME obtained by sonication and incorporated into the hydrogels studied. For AME, a previously described mild homogenization protocol was used (14). This method is based on a gentle approach designed to preserve the functionality and biological activity of the extracted proteins. In contrast, protein extraction from the AM tissue was obtained through Precellys (Bertin Technologies, Montagne-le-Bretonneux, France) homogenization of the AM extract in a buffer containing 7 M urea, 2 M thiourea and 4% CHAPS. The proteins obtained by the two methods were digested with trypsin (Sigma Aldrich, St. Louis, MO, USA) following the FASP protocol described previously (33). The trypsin was prepared in 50 mM ammonium bicarbonate and added to the protein at a trypsin: protein ratio of 1:10, incubating the mixture overnight (ON) at 37°C. The resulting peptides were dried in a RVC2 25 speedvac concentrator (Christ, Vienna, Austria), resuspended in 0.1% Formic Acid (FA) (Sigma Aldrich, St. Louis, MO, USA), desalted and again resuspended in 0.1% FA using C18 stage tips (Millipore, St. Louis, MO, USA). The peptides recovered were then loaded onto an EvoSep One (EvoSep, Odense, Denmark) chromatograph coupled on-line to a TIMS ToF Pro mass spectrometer (Bruker, Coventry, UK), using Parallel Accumulation Serial Fragmentation (PASEF) acquisition to rapidly achieve a highly sensitive analysis following the 30 SPD protocol (approx. 44 min runs) under default Evosep settings. Data-dependent acquisition (DDA) was used and the data was processed with MaxQuant software (Max Planck Institute of Biochemistry, Martinsried, Germany) using default parameters (34).

Searches were carried out against a database consisting of human protein entries from Uniprot/SWISSPROT [UniProt Consortium, European Bioinformatics Institute (EMBL-EBI), Hinxton, UK; Swiss Institute of Bioinformatics (SIB), Geneva, Switzerland; and Protein Information Resource (PIR), Georgetown University, Washington, DC, USA]. Carbamidomethylation of cysteines was considered as a fixed modification and the oxidation of methionines as a variable modification. Proteins passing a false detection rate (FDR) filter of <1% at the protein level were considered for further analysis. Spectral Count data was used to compare the different conditions, specifically using a modified spectral counting method named Normalized Spectral Abundance Factor (NSAF) (35). Briefly, protein spectral counts (the sum of all peptide identifications obtained for a given protein) were corrected by protein length, obtaining the Spectral Abundance Factor (SAF) for each protein. These SAF values were further normalized against the sum of all SAF values in a specific sample and expressed as a percentage of the total.

The mass spectrometry proteomics data have been deposited to the ProteomeXchange Consortium via the PRIDE (36) partner repository with the dataset identifier PXD055969.

Gene Ontology (GO) enrichment analysis was carried out using the DAVID online tool (http://david.abcc.ncifcrf.gov/summary.jsp, Accessed 7 October 2020) (37). DAVID is a GO Term annotation and enrichment analysis tool used to highlight the most relevant GO terms associated with a given gene list.

### 2.3 Hydrogel synthesis

Two hydrogels were synthesized, designated as hydrogel A (treatment 3) and B (treatment 4), each with distinct formulations but encapsulating the same AME proteins.

#### 2.3.1 Hydrogel A

**Figure 1:**
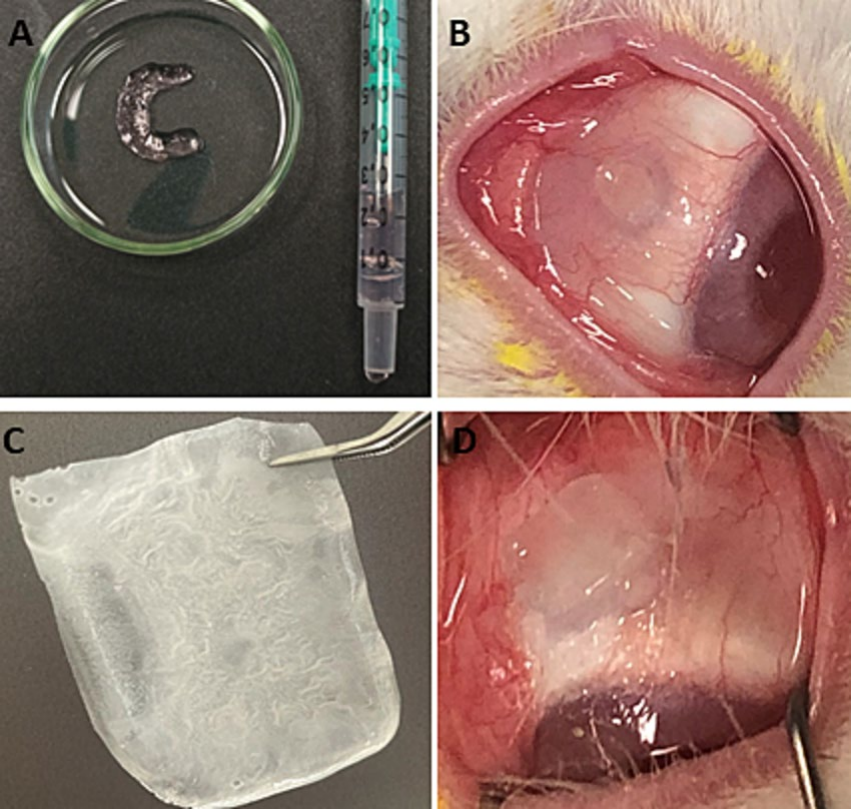
**(A)** Image of hydrogel A (ointment-like hydrogel) on a Petri dish. **(B)** Image of hydrogel A applied to the conjunctiva of a rabbit from group 3. **(C)** Image of hydrogel B (physically cross-linked hydrogel). **(D)** Image of rehydrated hydrogel B applied to the conjunctiva of a rabbit from group 4.

Hydrogel A is an injectable dynamic hydrogel prepared using HA-SH as described below (Figures 1A,B). Initially, HA-SH (20 mg) with a molecular weight (MW) of 250 kDa and with 3.6 μmol thiol groups (Haworks, USA) was dissolved in 500 μL of deionized water (solution A). Subsequently, solution B was obtained by diluting 3.6 μL of an aqueous solution of HAuCl4 (0.01 M, 99% purity: Aldrich, USA) in 686.4 μL of deionized water. Both solutions (A and B) were shielded under nitrogen to prevent oxidation of the free thiols until solution B was slowly added to solution A with continuous magnetic stirring. Phenol red (10 μL, 0.07%wt: Aldrich, USA) was introduced as a pH indicator and the pH of the hydrogel was adjusted to approximately 7.5 using an aqueous 5 M solution of NaOH (Aldrich, USA), after which 100 μL of 10X PBS (Merck, Germany) was added to buffer the samples. The remaining free thiolates were left as oxidized until no variation in the rheological properties (loss and storage moduli) were observed after approximately 48 h at 4°C. AM proteins were incorporated into the hydrogel during the buffering step, resulting in a final protein concentration of 1 mg/mL when determined using the Bradford assay (Thermo Fisher Scientific, USA).

#### 2.3.2 Hydrogel B

Hydrogel B, a physical hydrogel, was prepared as a film using different polymers in its formulation, all of them FDA approved (Figures 1C,D). Hydrogel B is composed of 50 kDa MW PVP, 100 kDa MW PVA, 2 kDa PEG, 125 kDa Eudragit S100, HA with a MW of 750 kDa and glycerol. The incorporation of HA to what was a previously described formulation was aimed at improving ocular mucoadhesion (38). Films were prepared using the solvent-casting technique, whereby PVA and PVP were mixed for 2 h in water at 90°C (39) and after cooling. PEG, Eudragit S100 and HA were added to the mixture, and the pH was adjusted to 7.4 using 10 mM PBS and NaOH/HCl 1 M. AM proteins were incorporated prior to evaporation of the solvent at a constant temperature of 37°C. Finally, the resulting film was cut into 0.4 × 0.4 cm fragments with an average thickness of 1 mm, each containing 1 mg/mL of AM protein.

### 2.4 Release of AM proteins from hydrogel A and B

The release of AME proteins from the hydrogels was evaluated in a similar manner in both cases. Following the encapsulation of the lyophilized AM extract, 50 μL of hydrogel A and a fragment of hydrogel B, designed in a format suitable for application on the conjunctiva with dimensions of 0.5 × 0.5 × 0.1 cm, were placed in a release system developed by our group and previously reported (39).

Both hydrogels were exposed to the external environment while a buffering agent was introduced at the upper end, flowing uniformly over the surface of the material. Aliquots of this buffer containing the proteins released from the hydrogels were subsequently collected. The hydrogels gradually dissolved in the buffered solution, mimicking the action of tears.

Despite the stabilization of hydrogel B through physical interactions, the introduced liquid was able to dissolve the polymers comprising the matrix, ultimately leading to the complete dissolution of the hydrogel. The liquid used was an isotonic saline solution for ocular irrigation, known as Balanced Salt Solution (BSS; AJL Ophthalmic, Spain). Since the natural tear flow rate in the human eye is 2.2 μL per minute, this value was scaled up by a factor of 50–110 μL per minute to meet the experimental requirements. The total release of proteins was completed after 360 min, and samples were collected every 15 min to study the release kinetics. Protein quantification was performed indirectly using EZQ fluorescence with a BioTek Synergy Neo 2 multimode reader and GEO 5 3.1 software. Subsequently, the values were interpolated using a calibration curve in the range of 1–25 μg/mL. Finally, the data obtained were processed using GraphPad Prism software.

### 2.5 Animal studies

This study was carried out on male New Zealand White rabbits (*n* = 44, weight 2.5–2.7 kg), with all the animal procedures adhering to the guidelines set forth by the Association for Research in Vision and Ophthalmology (ARVO) regarding the Use of Animals in Ophthalmic and Vision Research. Approval for the experimental protocols was obtained from the Animal Experimentation Ethics Committee of the Basque Public Health System and the Institute for Health Research Biogipuzkoa (permit number: CEEA20/15), as well as the Provincial Council of Gipuzkoa (Cod. PRO-AE-SS-196), ensuring compliance with both European and national legislation.

An animal model of alkali corneal burn was established as described previously (40). Briefly, rabbits were sedated with a subcutaneous injection of Meloxicam (0.2 mg/kg: Boehringer Ingelheim, Spain) and an intramuscular injection of ketamine/xylazine (35/20 mg/kg: Halmein Pharmaceuticals, Germany/Carlier, Spain), followed by anaesthesia with Sevofluorane 3% (Abbvie, Spain) and topical anesthesia with 2–3 drops of Colircusi Anestésico Doble® (Alcon-Cusí, Spain). An alkali burn was induced in the right eye of each rabbit by placing an 8 mm strip of filter paper soaked in 1 M NaOH on the central cornea for 60 s under an ophthalmic surgical microscope. Subsequently, the eyes were rinsed with 200 mL saline solution for 1 min, and eye drops containing tobramycin and dexamethasone phosphate (Alcon Cusi, Barcelona, Spain) were administered post-surgery (p.s.) to each animal. The rabbits were then randomly divided into four groups (*n* = 11 per group) and they received one of the following treatments: group 1 (no experimental treatment), post-surgical eye drops alone; group 2, Amniotic Membrane Transplantation (AMT); group 3, dynamic hydrogel (hydrogel A); and group 4, physically cross-linked ocular insert hydrogel (hydrogel B). Both hydrogels were loaded with the AME and the contralateral uninjured eye (native) was used as the control.

After inducing the corneal lesion, hydrogels A and B were placed in the conjunctival sac following different approaches. Hydrogel A was administered to rabbits in Group 3 via a syringe in gel format, spread over the entire corneal surface by blinking. Hydrogel B was pre-hydrated for 60 s in a Balanced Salt Solution (BSS) before it was placed in the upper conjunctival sac of Group 4 rabbits, applying gentle pressure to enhance gel adhesion. Hydrogels were administered every 12 h over 7 days by an experienced ophthalmologist. After inducing the corneal wound, the AM attached to the cornea as a monolayer in Group 2 (41) was removed on the 7th day of follow-up following administration of 2–3 drops of topical anesthesia.

### 2.6 Clinical examination

Corneal wound healing was monitored by capturing photographs (EOS 750D, Canon Inc., Japan) immediately post-surgery (day 0) and on days 7, 14, 21 and 28 p.s. The images were obtained under cobalt blue light post 0.1% fluorescein sodium staining and the area of the corneal epithelial lesion was quantified (mm^2^) three times using Image J software (National Institutes of Health (NIH), Bethesda, MD, USA). Technical replicates were employed for the analysis of the de-epithelialized area, with three measurements performed for each biological replicate. The biological replicates consisted of nine specimens per group.

### 2.7 Histopathological examination

Two rabbits were selected randomly from each group and sacrificed with an overdose of anesthesia on day 2 post-alkali burn (acute healing), while the remaining animals were sacrificed on day 28 (late healing). The corneas were harvested, fixed in 4% paraformaldehyde, preserved in 30% sucrose, embedded in OCT to obtain cryostat sections (7 μm). The sections were stained with hematoxylin and eosin (H&E) or Masson trichrome and random fields of the injured area and the adjacent tissue were analyzed. Images were obtained under a light microscope (Nikon Eclipse 80i, Japan), and the epithelial and stromal thicknesses were measured at three random locations in each field using ImageJ software. The presence or absence of an endothelial layer and inflammatory cell density were recorded.

### 2.8 Statistical analysis

The data were analyzed using SPSS 22 software (IBM-SPSS, USA). In the analysis of the *in vivo* studies, and given the sample size and the non-normal distribution of the variables, non-parametric tests were used (Mann–Whitney U, Kruskal Wallis and Chi-squared). Variables with a normal distribution, such as the stromal and epithelial thickness, and their relationship with endothelial damage, were studied using parametric tests (ANOVA and Student T-test). Significance was set at *p* < 0.05.

## 3 Results

### 3.1 Proteomic characterization of the AM

**Figure 2:**
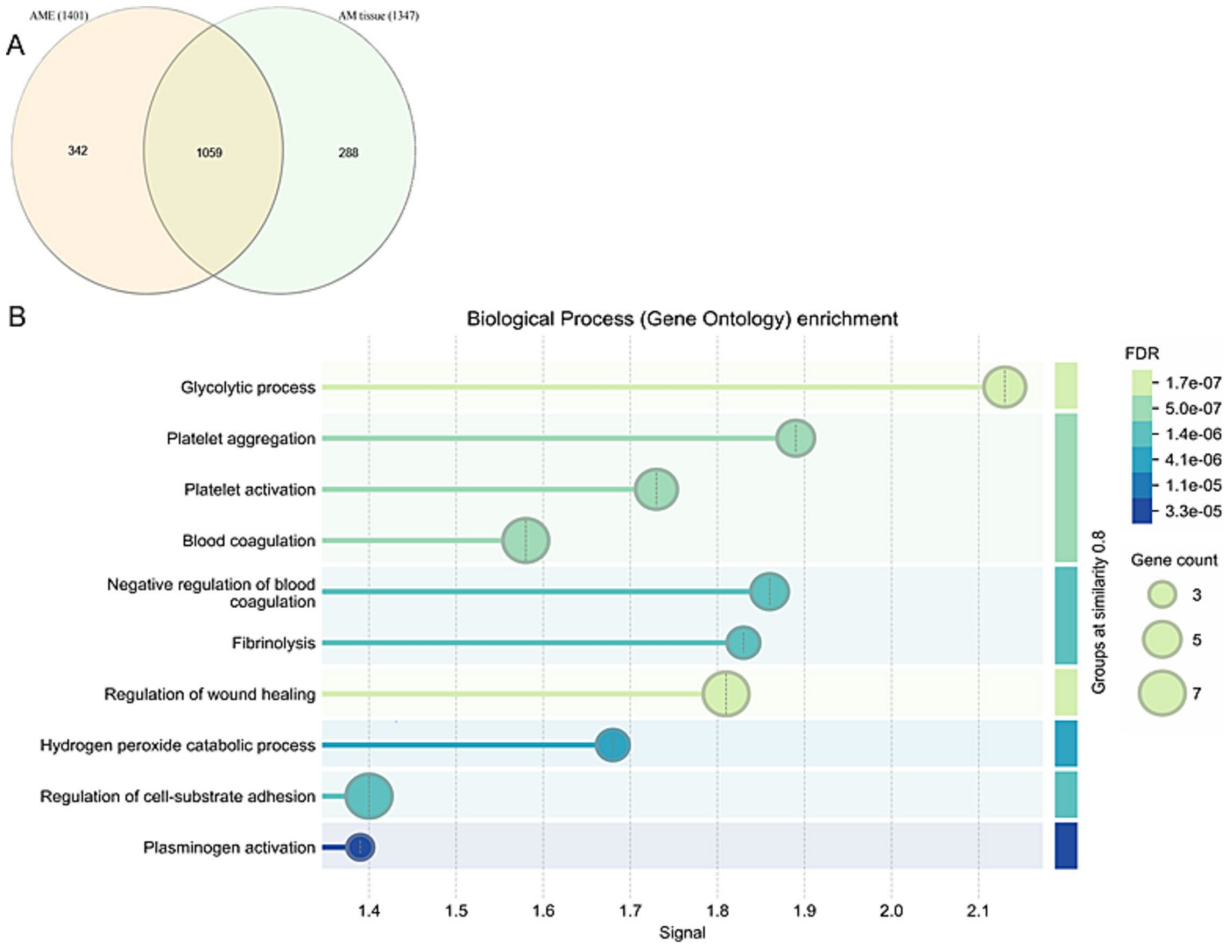
**(A)** Venn diagram representing exclusive and mutually inclusive proteins between AM proteins extract (AME) and AM tissue. **(B)** Gene Ontology (GO) analysis was performed to characterize the functional processes associated with the proteins identified in both the AME and AM tissues.

The proteins in the AM tissue (group 2) and the AME introduced into the hydrogels (groups 3 and 4) were analyzed, identifying a total of 1,689 proteins. Of these, 1,059 were common to both the tissue and the extract, with 288 proteins (17%) identified exclusively in the AM tissue and 342 proteins (20%) specific to the AME (Figure 2A and Supplementary File S1 for the complete list of proteins in these formulations). The difference in composition may be attributed to the different protein extraction protocols used in this study. For protein extraction from the AM, a gentle homogenization protocol was employed, based on a non-aggressive approach designed to preserve the functionality and biological activity of the extracted proteins. In contrast, protein extraction from the AM tissue was performed using a Precellys device and a buffer containing 7 M urea, 2 M thiourea, and 4% CHAPS. This latter protocol was designed to obtain a broader spectrum of proteins, including those that are insoluble or membrane-associated.

A GO analysis was carried out to characterize the functional events in which the proteins identified in both the AME and the AM tissue are involved (Figure 2B). These processes can be mainly grouped as: (i) Cellular activity and wound healing consisting of cell adhesion, extracellular matrix remodeling and protein synthesis; (ii) Immune responses, combining the GO terms of innate immune responses, complement activation (classical pathway), complement activation and inflammatory responses; and (iii) Platelet aggregation, comprising the GO terms of blood coagulation, platelet activation and homotypic cell–cell adhesion.

### 3.2 AM protein release from the hydrogels

**Figure 3:**
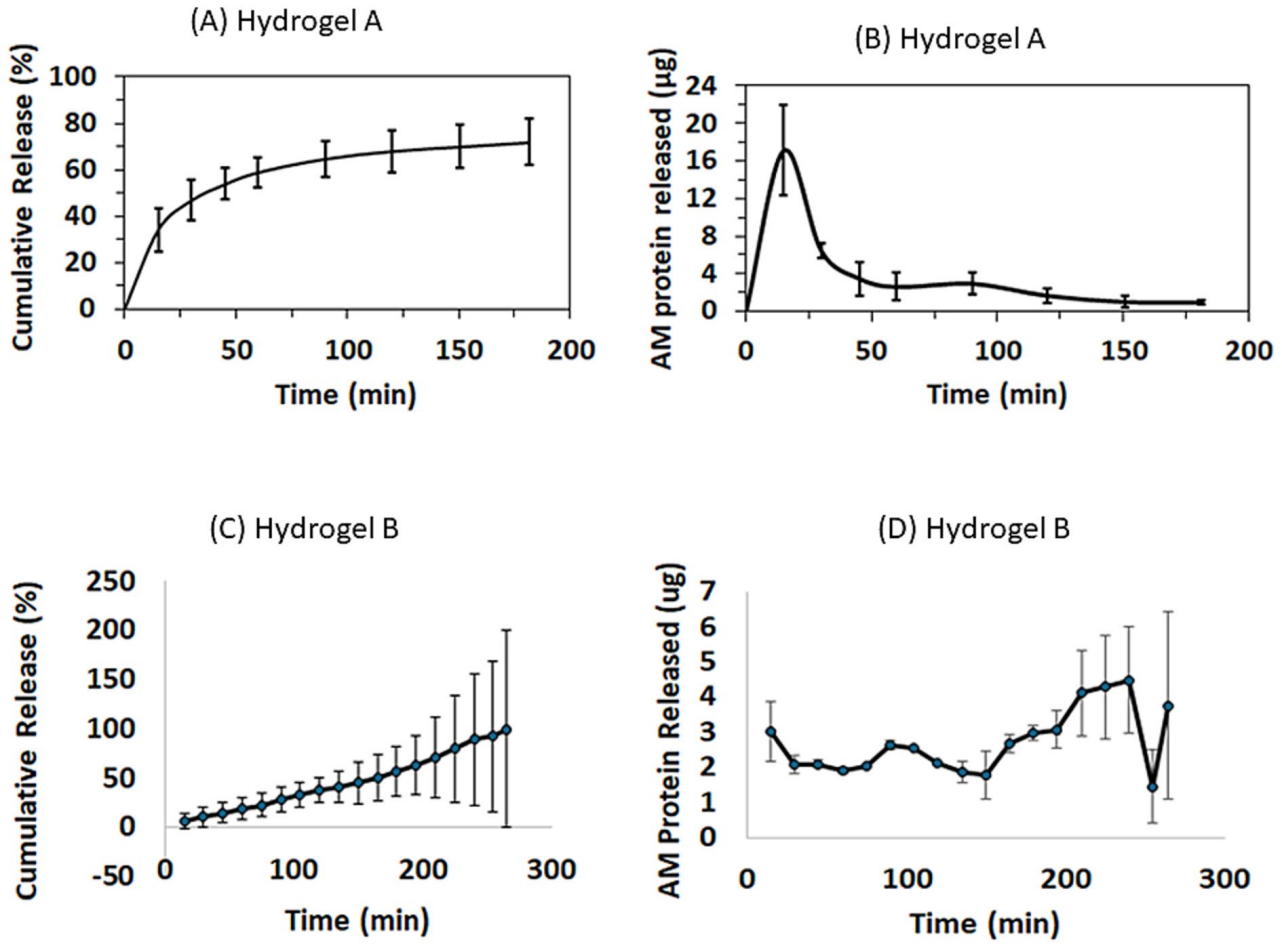
Release kinetics of AM proteins from Hydrogel A and Hydrogel B. **(A)** Cumulative release and **(B)** mass of AM protein released over time (μg) for Hydrogel A. **(C)** Cumulative release and **(D)** mass of AM protein released over time (μg) for Hydrogel B.

When evaluating the release kinetics of AM proteins from hydrogel A, it was observed that 34 ± 9% of the total protein content was rapidly released within the first 15 min (Figure 3A). Subsequently, the protein concentration in the collected BSS medium gradually decreased over time, eventually falling below the assay’s detection threshold after 60 min. At this point, the cumulative protein release reached approximately 60%, corresponding to a release duration equivalent to 2 days when considering the natural tear flow rate in the human eye. These findings suggest that AM proteins are retained within hydrogel A via weak physical interactions, distinct from the dynamic bonds observed in hydrogel B, which influence the hydrogels’ solubility in BSS during the experiment. The pronounced release of proteins during the first hour of the experiment (up to 30 ± 3 μg/mL, Figure 3B) is particularly significant for ocular regeneration. This initial burst release of AM proteins could potentially facilitate rapid activation of the healing process in the damaged area, while the subsequent sustained release would help maintain this therapeutic activity over an extended period.

Hydrogel B gradually dissolved into the serum, releasing both the AM proteins and the polymers constituting the hydrogel. The total amount of released proteins was quantified every 15 min, showing that 50% of the total protein content was released after 165 min, 80% after 225 min, and 100% after 265 min (Figure 3C). The maximum protein release occurred at 241 min (Figure 3D). This sustained release highlights the material’s ability to ensure a consistent flow of proteins into the animal’s eye.

### 3.3 Clinical data

In this study, 44 rabbits were distributed evenly into four groups and a corneal lesion was induced in each one with 1 M NaOH as indicated in the Methods. Of these, eight rabbits were sacrificed on day 2 p.s. and 36 rabbits on day 28 p.s. to perform histological analyses (see section 3.3). While one of the four study groups did not receive any experimental treatment, Group 2 received AMT applied directly to the eye, whereas Groups 3 and 4 received the AME delivered through hydrogels. The ointment-like consistency of hydrogel A, combined with the mucoadhesive properties of HA-SH, facilitated its uniform spread across the cornea. By contrast, the physical crosslinking in hydrogel B conferred this matrix with remarkable stability and good mucoadhesion.

Following the induction of corneal wounds, Hydrogel A was applied to Group 3 animals every 12 h for 7 days in the upper conjunctival sac. The hydrogels were safely administered via syringe and demonstrated biocompatibility in the lower fornix. Daily general observations indicated that 6 h post-application, the hydrogel was no longer visible on the ocular surface, having completely degraded. After 12 h, no traces of the hydrogel were detected.

Similarly, after inducing corneal wounds, Hydrogel B was applied to Group 4 animals every 12 h for 7 days in the upper conjunctival sac, with gentle pressure applied to enhance gel adhesion. Observations revealed that the hydrogels were safely retained and showed biocompatibility in the upper fornix. Daily evaluations of the films demonstrated that, while the films exhibited mild wear 6 h post-application, they did not degrade or dissolve entirely. By the 12-h observation, no hydrogel remnants were detected.

In both cases, no signs of irritation, redness, or swelling of the eyes or ocular structures were observed in any animal as a result of hydrogel application. The animals were monitored and assessed over the 7-day application period to detect any potential signs of pathology.

**Figure 4:**
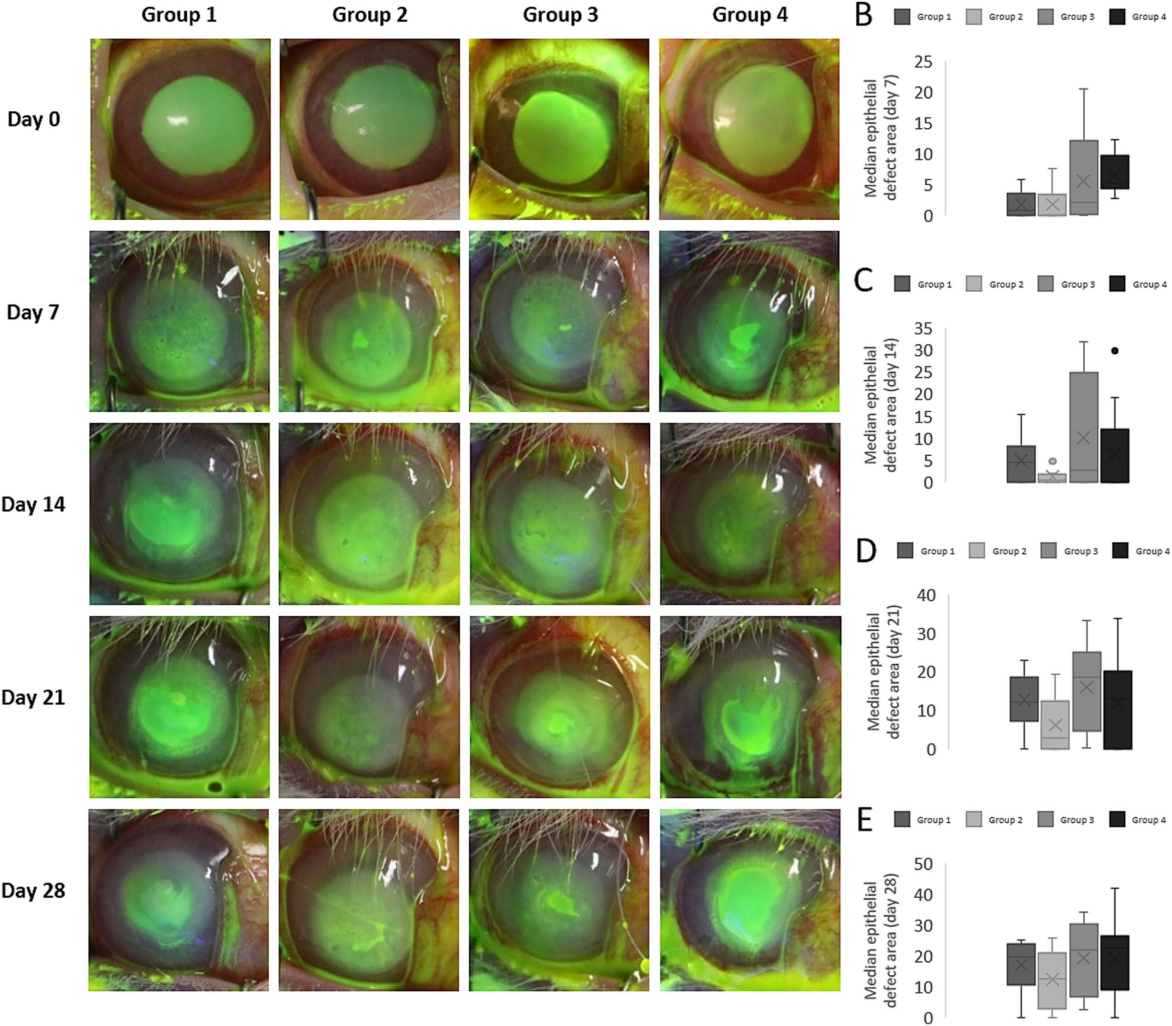
Evolution of the epithelial lesion in rabbit eyes. **(A)** The epithelial defect was monitored by fluorescein staining and a study of the lesion in each group showed significant wound healing on each day studied relative to the day of surgery. The wound area decreased until day 14 post-surgery (p.s.), after which reopening of the wounds was observed that possibly reflected the intensity of the alkaline burn and the fragility of the newly formed epithelium. **(B–E)** Median epithelial defect area (mm^2^) on day 7 **(B)**, 14 **(C)**, 21 **(D)**, and 28 **(E)** p.s. The asterisks indicate significant differences relative to the control group (*p* < 0.05).

The progression of epithelial defects was monitored for 28 days using fluorescein staining (Figure 4A) and a reduction in the epithelial lesion area (mm^2^) was observed in all the treatment groups on days 7, 14, 21, and 28 p.s. relative to the day of intervention (*p* < 0.005, Wilcoxon test: Table 1). A high incidence of persistent epithelial defects and wound reopening was observed in 91.6% of corneas, probably reflecting the intensity of the alkaline burn and the fragility of the newly formed epithelial (42). There were statistically significant differences in the area of the epithelial defect between the groups on day 7 (*p* = 0.014, Kruskal-Wallis test), with significant differences between group 1 and group 4 (*p* = 0.02) and between group 2 and group 4 (*p* = 0.006, Mann–Whitney *U* test). However, there were no significant differences between the groups on days 14, 21 and 28 (Kruskal–Wallis test: Figures 4C–E). Hydrogel A facilitated the fastest healing, while eyes treated with hydrogel B exhibited delayed wound repair. Thus, the differences between the untreated eyes and those that received hydrogel B were only significant on day 7 p.s., with open wounds evident in all eyes (Figure 4B).

**Table 1:** Median and Interquartile range (IQR) of the areas carrying epithelial defects (mm^2^) in each group on the day of surgery and on days 7, 14, 21, and 28 post-surgery.

Group	Day 0	Day 7	Day 14	Day 21	Day 28
1	54.75 (50.21–60.69)	0.78* (0.00-3.44)	4.36* (0.00-8.03)	11.96* (7.18-18.45)	19.57* (10.52-23.75)
2	54.62 (51.91–60.14)	0.00* (0.00-3.32)	0.44* (0.00-1.63)	2.65* (0.00-12.23)	12.51* (2.63-20.81)
3	52.82 (49.62–58.41)	1.95* (0.16-12.05)	2.57* (0.00-24.77)	18.53* (4.45-25.10)	21.85* (6.70-30.32)
4	57.46 (56.50–62.12)	5.04* (4.27-9.66)	0.00* (0.00-11.93)	12.72* (0.00-20.06)	22.42* (8.89-26.43)

* Significant difference relative to day 0, *p*-value < 0.05.

**Figure 5:**
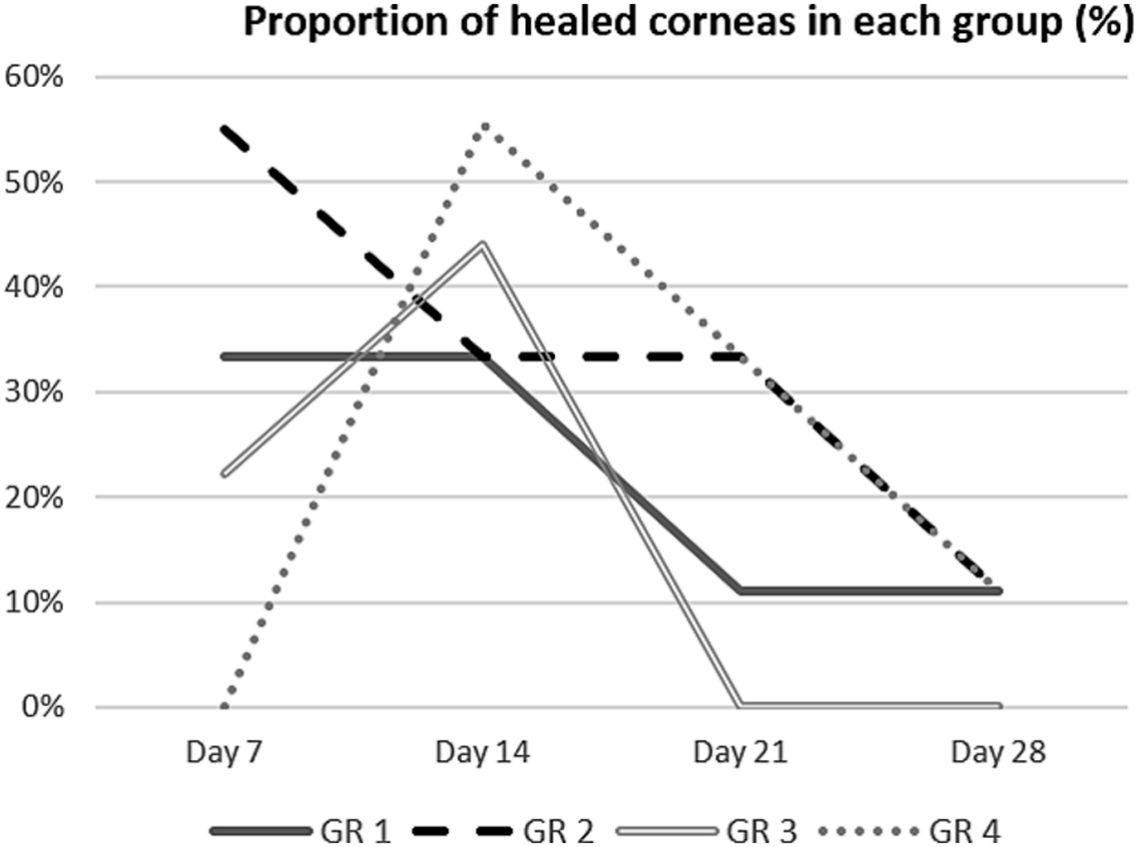
Proportion of fully healed corneas (percentage) in each group on days 7, 14, 21, and 28 post-surgery.

The proportion of completely healed corneas did not differ significantly between the untreated and treated eyes on the days studied (Figure 5). On day 14 p.s., the proportion of completely healed corneas was higher in group 3 (44.4%) and 4 (55.5%) than in the untreated eyes in group 1 (33.3%). The enhanced healing following these treatments, even after the treatment was discontinued on day 7 p.s., suggests that AM proteins may enhance the induction of wound closure in the early stages of healing.

### 3.4 Histological analysis

**Figure 6:**
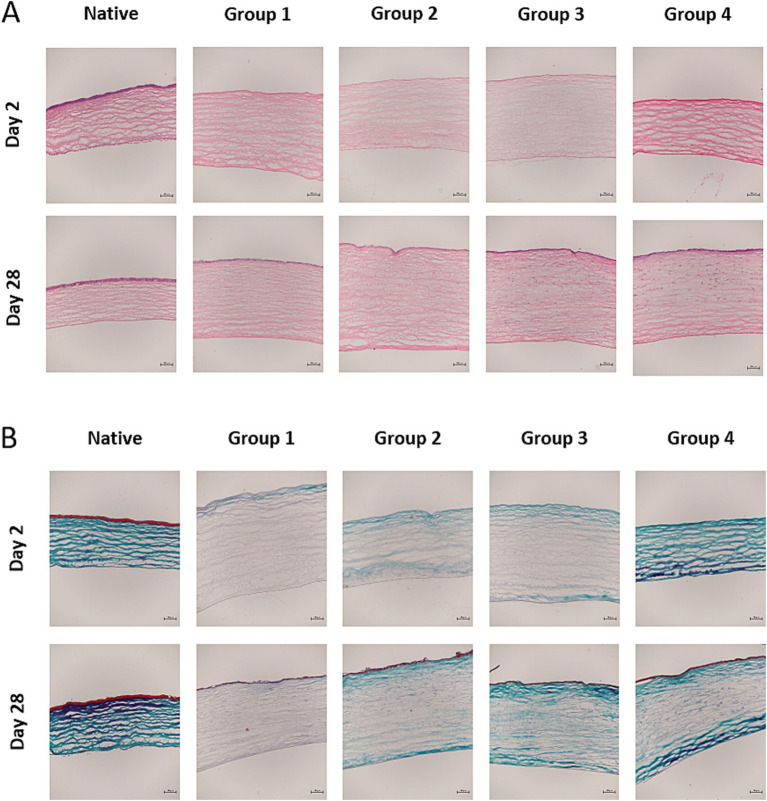
**(A)** Haematoxylin–eosin stained central sections on days 2 and 28 post-surgery (p.s.: 10x). In the early healing stage, the burned corneas exhibited stromal thickening, endothelial damage, a lack of epithelial regeneration, a keratocyte deficit in the corneal stroma and scant inflammatory cell infiltration. This together reflects the severity of the lesion provoked in the animal model. At a late healing stage (day 28 p.s.), stromal thickening persisted with a separation of collagen fibers in all the wounded corneas. There were no significant differences in the thickness of the layers analyzed between the groups, although the oedema in the central cornea was smaller in groups 2 and 4. **(B)** Masson trichrome stained central sections on days 2 and 28 p.s. (10x). Severe alterations to collagen staining were evident on day 2 p.s. that persisted to some extent on day 28 p.s. Scale bars 200 μm.

The histological analysis focused on the stromal, epithelial and endothelial layers, as well as assessing the presence of inflammatory cells during the early and late healing phases (days 2 and 28 p.s., respectively). No significant differences were observed in the stromal and epithelial thickness between groups (ANOVA test), despite the apparent increase in epithelial thickness in the eyes of group 2 animals on the days analyzed (Table 2). Central corneal sections exhibited a thicker stroma and thinner epithelium in wound sections relative to the native corneas (Figure 6A). Masson’s trichrome staining highlighted severe alterations to the collagen staining that persisted from the early to the late healing phases (Figure 6B).

**Table 2:** Stromal and epithelial thickness (μm, mean ± SD) in the central and lateral corneal sections on days 2 and 28 p.s. in each group, and in the native cornea.

Section	Day	Layer	Group 1	Group 2	Group 3	Group 4	NC
C	2	S	1,301 ± 261	1,032 ± 185	1,308 ± 155	1,024 ± 280	689 ± 96
C	2	E	0 ± 0	2.79 ± 3.95	0 ± 0	0 ± 0	17.08 ± 4.13
L	2	S	1,311 ± 257	935 ± 52	1,279 ± 84	1,288 ± 643	668 ± 117
L	2	E	1.87 ± 2.65	5.55 ± 5.04	2.33 ± 3.30	2.56 ± 3.63	18.98 ± 2.15
C	28	S	1,291 ± 115	1,280 ± 237	1,268 ± 179	1,125 ± 170	668 ± 79
C	28	E	6.14 ± 1.94	7.27 ± 5.90	5.85 ± 2.95	6.47 ± 4.89	16.58 ± 1.79
L	28	S	1,120 ± 293	1,391 ± 281	1,272 ± 255	1,371 ± 281	699 ± 133
L	28	E	8.92 ± 1.10	11.01 ± 2.79	8.42 ± 3.00	8.25 ± 3.28	17.35 ± 1.74

C, central section; L, lateral section; E, Epithelium; S, Stroma; NC, native cornea.

**Figure 7:**
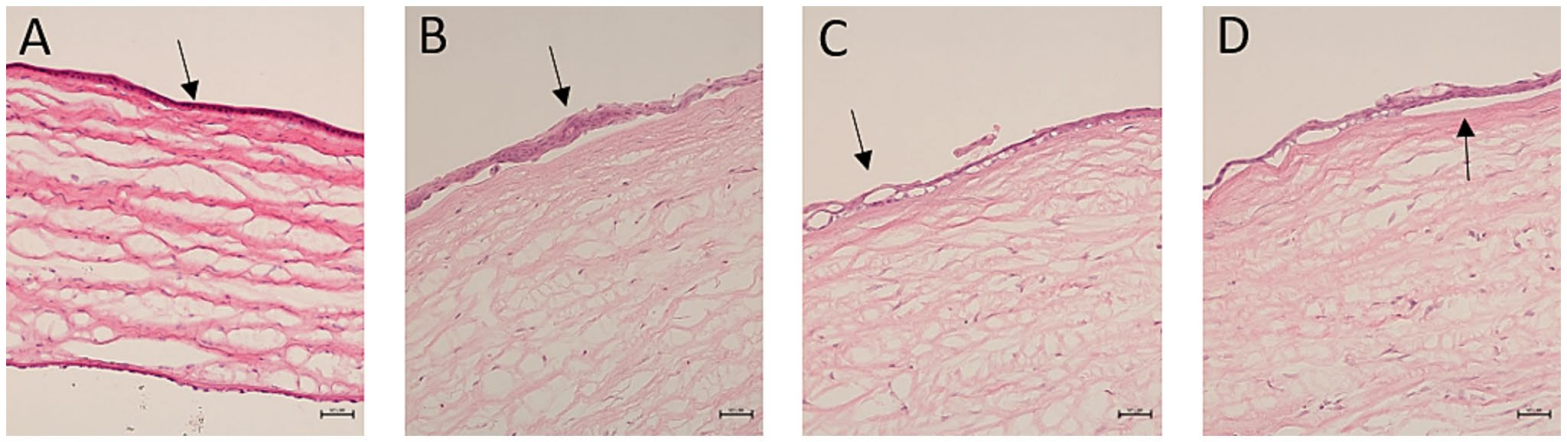
Hematoxylin–eosin stained corneal sections showing abnormal epithelial regeneration (20x). **(A)** The arrow indicates epithelial hypoplasia with monolayer cuboid cells; **(B)** the arrow indicates focal hyperplasia; **(C)** the arrow indicates a failure of the epithelial basal layer to compact; **(D)** the arrow indicates complete separation of the epithelial layer. Scale bars 100 μm.

**Figure 8:**
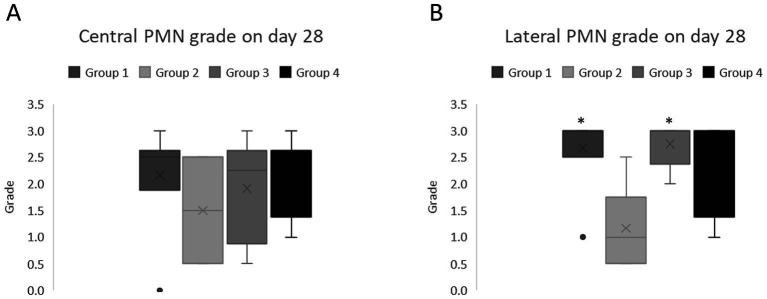
Median and Interquartile Range (IQR) of the inflammatory infiltration in the corneal sections on day 28 post-surgery. **(A)** Central sections; **(B)** lateral sections. The asterisks indicate significant differences relative to group 2 (AMT, *p* < 0.05: Kruskal Wallis test).

Abnormal epithelial regeneration was evident, with disrupted junctions between the epithelial layer and basement membrane observed (Figure 7). Fewer cells were present in the anterior stromal third on day 2 p.s. relative to day 28 p.s., with significant differences in the presence of inflammatory cells on day 28 p.s. between the eyes from group 2 animals relative to those from groups 1 and 3 (*p* = 0.01, Kruskal Wallis Test: Figure 8). Moreover, alterations to the endothelium were noted, with more extensive endothelial damage correlating with a significantly thinner epithelium (*p* = 0.001, Student *T*-test).

## 4 Discussion

Hydrogels represent a versatile platform in the realm of personalized and precision medicine, offering a range of functionalities. Moreover, the emergence of new biomaterials promises to overcome some of the limitations associated with existing therapeutic modalities. Hence, here we explore two distinct hydrogels as biocompatible matrices for the controlled release of AM proteins, with the aim of improving wound healing in response to severe, acute corneal lesions. Given that the nature of the hydrogel conditions the release of any of its components, two types of hydrogels were considered here: a state-of-the-art hydrogel based on dynamic bonds and another based on the physical binding of different biocompatible biopolymers. While the former is administered in the form of an ointment-like hydrogel, designed to ensure rapid release of any active ingredient, the latter is applied in the form of a film to ensure sustained release over time. In both of these cases, the active ingredient used here was a cocktail of proteins extracted from human AM tissue (AME).

The regenerative nature of proteins found in the AM has been studied widely (5, 43, 44) and as such, the protein profile of the AME obtained through AM sonication was analyzed to ensure that the majority of the proteins present in the AM were preserved in the AME. The proteomic analysis demonstrated that the protein composition of the AME was very similar to that of the AM tissue, sharing 62% of the proteins. The biological processes mediated by these proteins underscore their significant role in wound healing. These processes include (A) cell adhesion, (B) extracellular matrix (ECM) remodeling, (C) protein synthesis, (D) innate immune response, (E) plasminogen activation, (F) inflammatory responses, (G) blood coagulation, (H) platelet activation, and (I) homotypic cell–cell adhesion. Proteomic analyses have demonstrated that both the amniotic membrane (AM) as a tissue and its protein extracts preserve ECM proteins such as collagens, glycoproteins, proteoglycans, metalloproteinases, and growth factors, as previously described (45). These ECM proteins contribute to tensile strength, provide structural support, stabilize basal membrane components, and regulate fibrillary collagen growth.

Additionally, ECM glycoproteins—including fibrillin, fibrinogen, laminin, and vitronectin—were identified, along with growth factors such as transforming growth factor-beta (TGF-*β*) and epidermal growth factor (EGF), both of which are known to play pivotal roles in tissue regeneration and wound healing (46, 47). Proteins involved in coagulation pathways, platelet activation, and aggregation, which are directly linked to wound healing, were also identified. Pathways related to ATP synthesis, vascular endothelial growth factor (VEGF) signaling, the plasminogen activation cascade, glycolysis, and cytoskeletal regulation were associated with essential processes such as angiogenesis, collagen deposition, and epithelialization. Angiogenesis, the formation of new blood vessels from pre-existing ones, is vital for wound healing as it ensures the delivery of oxygen, nutrients, and growth factors to the injury site (48). The plasminogen activation pathway, specifically, is critical for breaking down blood clots and facilitating ECM remodeling, both of which are key to efficient wound repair. Controlled clot degradation prevents pathological scarring, while ECM remodeling supports cell migration, tissue restructuring, and regeneration (49).

The effectiveness of the two synthesized hydrogels was evaluated *in vivo* in a well-established model of an alkali corneal burn in albino rabbits. Various techniques exist to induce corneal lesions, each with its own advantages and challenges. For example, lesions induced by physical methods tend to exhibit rapid closure rates, making it difficult to discern significant differences between therapeutic interventions (50). Conversely, alkali burns with agents like NaOH produce more severe and persistent epithelial defects, mimicking clinical scenarios of severe corneal burns (51). Here, we opted to use an aggressive wound model involving the entire corneal layer, akin to scenarios warranting AMT in clinical practice (42). However, despite efforts to standardize the alkali burn technique (42), considerable heterogeneity persists in the literature regarding lesion size, NaOH concentration and the duration of exposure, complicating direct comparisons between studies (52–54).

The alkali burn model employed involved applying NaOH (1 M) for 60 s, which required approximately 3–7 days to achieve complete wound closure, enabling the effects of the hydrogels to be assessed over a 7-day period (40, 55). However, as the AMT was sutured onto the cornea, the initial measurements of fluorescein staining were taken from day 7 p.s. onward. In accordance with previous models, the initial closure phases were rapid in this model, making inter-group differences challenging to detect in the first week. Despite the aggressive nature of our model, no significant differences in epithelial defects were observed between the experimental groups on days 0, 14, 21, or 28 p.s., although subtle differences were noted between the control and hydrogel-treated corneas, as discussed below.

Corneas treated with AMT exhibited better epithelial defect outcomes relative to the untreated controls, even after AMT removal on day 7 p.s. Hence, AM proteins appear to exert a crucial influence on the early healing phases, possibly modulating wound closure pathways beyond the removal of the AMT (56, 57). Differences between the completely healed corneas on day 14 p.s. may reflect the proteins secreted in earlier phases, influenced by biomaterial presentation. The ointment-like formulation of hydrogel A facilitates contact but it may be associated with limited protein release when compared to the more solid formulations due to the more rapid clearing of the latter (58). Extended ointment application beyond day 7 p.s. might yield improved outcomes. Hydrogel B initially delayed epithelialization, possibly due to its mobile nature as it has been postulated that direct contact of the mobile hydrogel with the regenerating epithelium may have a negative effect on wound closure (55). However, subsequent improvements and stronger healing on days 14 and 21 p.s. suggest positive effects once these effects of friction have ceased.

The histological examination of epithelial thickness agreed well with the relative wound closure area. The aggressive nature of the model impeded epithelial regeneration and although AMT better restored epithelial thickness, there were only minimal differences with the other Groups (1, 3, and 4). Similarly, recent publications indicated that the full thickness of the epithelial layer could be recovered after chemical burns (51). There was little basal layer compaction with separation of the epithelial layer in some cases and this failure of the newly formed epithelium to bind to the basal layer could be related to the reopening of wounds *in vivo*. Histological studies to track the persistent epithelial defects indicated that reopening could occur after the healing associated with direct contact and in such cases, alterations were observed at the level of the epithelial layer and basal membrane. Indeed, intraepithelial pseudocysts have been described and displacement or duplication of Bowman’s membrane in the epithelium of human corneas has been observed (59). Furthermore, fibrous material has been seen to accumulate between the basement membrane and the epithelium (60). Similarly, *in vivo* studies using the same burn model described the absence of a basement membrane between the epithelium and stroma, as well as an increase in the intercellular spaces between the regenerated epithelial cells (54). This could explain by the reopening of the wound in all the burned corneas, especially in those in which the ocular hydrogel insert was employed, the mobility of which could explain the size of the epithelial lesion on day 7 p.s. that closed after removal of the film.

Consistent with previous studies, few cells were observed during the early phases of healing across most samples, particularly in the central regions. Notably, there was an absence of epithelial and endothelial layer formation, as well as a significant depletion of stromal cells in large areas of the stroma, although inflammatory cells were present. These findings align with prior reports indicating that the depth of alkali burns can extend to the endothelial layer, with damage to this layer observed in the same animal model (61). Similarly, other studies have demonstrated that immediately following alkali-induced damage, the majority of keratocytes are lost, becoming undetectable under light microscopy for up to 2 days post-injury (62). Furthermore, injuries caused by caustic agents such as NaOH are known to result in more severe tissue damage compared to physical agents. These injuries frequently serve as models for persistent epithelial defects, often characterized by recurrent wound reopening during follow-up periods (50, 51, 54). However, corneal cellularity recovered at all levels in the late healing phase, and although normal architecture was not restored, stromal thickening, inflammatory cell infiltration and an abnormal epithelium was achieved (51). Significant differences were evident in terms of inflammatory cell infiltration following AMT, possibly related to the known anti-inflammatory properties of this tissue (63). In this regard, we assume that the AM proteins delivered by hydrogel B (ocular insert hydrogel) will remain at the ocular surface for longer and hence, fewer inflammatory cells would be present than in the control eyes and following treatment with the hydrogel A ointment. Nevertheless, it must be borne in mind that the samples were obtained 21 days after the last contact of the proteins with the wound, which could have attenuated the effect of these proteins on corneal healing.

In conclusion, this study evaluated two promising hydrogels designed to gradually biodegrade and deliver sustained AM protein release to the corneal surface for over 7 days, with the aim of promoting corneal wound healing while minimizing irritation. The results indicate that these hydrogels can aid in the closure of caustic corneal wounds, producing more complete epithelialization and smaller lesions compared to untreated controls. Despite some limitations, such as insufficient mucoadhesion leading to de-epithelialization in the film-type hydrogel (hydrogel B), the study suggests that these hydrogels show potential as less invasive alternatives to AMT. They may offer a non-inferior option for promoting corneal wound healing, delivering similar benefits without requiring a more invasive surgical approach, thus lowering healthcare costs and simplifying patient management.

## Data Availability

The original contributions presented in the study are included in the article/Supplementary material. The mass spectrometry proteomics data have been deposited to the ProteomeXchange Consortium https://proteomecentral.proteomexchange.org/ with identifier PXD055969.
